# A Commentary on “A New Initiative on Precision Medicine”

**DOI:** 10.3389/fpsyt.2015.00088

**Published:** 2015-06-08

**Authors:** Udi E. Ghitza

**Affiliations:** ^1^Center for the Clinical Trials Network, National Institute on Drug Abuse, National Institutes of Health, Bethesda, MD, USA

**Keywords:** substance use disorders, alcohol, marijuana, cannabis, nicotine, tobacco

## Commentary

U.S. President Barack Obama recently announced a new Precision Medicine Initiative, and Drs. Francis Collins and Harold Varmus have begun to provide a vision for how some of this initiative might be implemented by the U.S. National Institutes of Health (NIH) (1). Precision medicine may be defined as “an emerging approach for disease treatment and prevention that takes into account individual variability in genes, environment, and lifestyle for each person” (2). A vision of the NIH portion of the Precision Medicine Initiative is to launch a large-scale national cohort study of a Million or more Americans to advance understanding of how to optimize treatments customized to individual variability in genomic and environmental health-determinants (2). A precision-medicine approach, using shared-decision making with patients and their providers as partners in patient-centered care, offers an important opportunity to improve substance-use disorders (SUDs) prevention and treatment outcomes (3, 4). Pertinent to precision medicine, the Collaborative Research on Addictions at NIH, comprising the National Institute on Drug Abuse, National Institute on Alcohol Abuse and Alcoholism, and the National Cancer Institute, in partnership with other NIH Institutes, Centers and Offices, is currently planning to launch a longitudinal cohort study of Adolescent Brain and Cognitive Development (ABCD). This study will follow 10,000 youth over up to a 10-year period, approximately ages 9–10 at baseline when largely naïve to use of alcohol, marijuana, nicotine, and other drugs. This national cohort study presents a key opportunity to answer fundamentally important questions to informing a precision-medicine approach regarding prevention of SUDs in youth (5). Several relevant questions are: (1) how does repeated exposure to abused substances, such as nicotine, alcohol, and cannabis, impact normative brain development essential for memory and cognitive functioning? (2) How do drug-altered brain-maturation pathways inform precision-medicine-tailored SUDs prevention approaches targeting high-risk youth? (3) Which brain-development events altered following adolescent drug use heighten likelihood of transformation of unhealthy drug use into full-blown SUDs in subpopulations with, or without, co-occurring mental health disorders? (4) How do drug-induced alterations in brain-development and memory impairments interact with genomic and epigenetic risk factors in these different subpopulations to increase vulnerability to SUDs? (5) In what manner does use of specific substances impact use of other substances? Thus, the objective of the ABCD study is timely to precision medicine: to better understand how exposure to abused substances modifies brain-development trajectories and how this relates to emotional and mental health, social development, memory and other cognitive function, as well as academic and other outcomes (5).

Numerous studies suggest that heavy substance use during childhood and adolescence influences long-term brain and cognitive development and heightens risks for SUDs and co-occurring mental disorders (6–9). Therefore, it is critical to recruit youth in the early, pre-symptomatic phase in order to measure mental health and psychosocial factors over time to understand how they contribute to observed changes in brain and cognitive development (5).To inform how clinicians may optimally intervene early to prevent escalation of unhealthy drug use in youth, this research will prospectively identify and characterize developmental processes across behavioral, cognitive, and neurobiological domains that give rise to transitions between hazardous substance use and SUDs trajectories in diverse populations of youth. Such longitudinal research will also evaluate how critical factors mediate or modify these relationships during sensitive brain-development windows. In such a large-scale longitudinal cohort study, an important consideration will be to implement a sampling strategy which includes a community-based sample that is broadly representative of the U.S. general population. Biospecimens will also be collected for subsequent genomic/epigenomic and other analyses in future research studies.

The ABCD study will leverage latest brain imaging advances, bioinformatics methods for analyzing biomedical big data, and electronic health records information to determine how substance use affects brain-development trajectories, relevant gene-environment interactions, memory capabilities, mental disorders, and other medical and functional outcomes. Another consideration is achieving sufficient statistical power and comprehensive controls to account for the many possible confounds in which youth who choose to frequently use alcohol or other drugs might also have other co-occurring problems either naturally or due to other lifestyle choices or circumstances. The ABCD study will also carefully characterize and control for socio-demographic, prenatal drug exposure, drug availability, family history, physical or sexual abuse, head trauma, behavioral, and other environmental risk factors (5).

Open data sharing and safeguarding privacy need to be cornerstones for such lines of research, to build a trustworthy scientific knowledge base and support a national network of scientists with innovative precision-medicine approaches to SUDs prevention and treatment. Collected genetic biospecimens need to be appropriately paired with other relevant health information and suitably processed, curated, and stored, in a manner whereby informed consent is obtained consistent with allowing participants’ permission for their future research use. Furthermore, to maintain high-quality repositories of biomedical big data, such research areas would need to develop sustainable operational and governance standards and conform to industry best practices (10). Moreover, to permit data sharing, procedures need to be put in place to enable harmonization of data collection, querying, extraction, and storage, across study sites with disparate electronic-health-record-system standards and data structures. Standardization of collected measures and data harmonization is needed to return clinical data in a consistent manner to a centralized repository and permit semantic mapping to achieve health information interoperability. The above research directions require a collaborative, sustained national effort involving many scientists, clinicians, and bioinformatics experts.

In summary, the ABCD study and similar research offer a valuable opportunity to inform precision-medicine research on how to leverage bioinformatics advances in genomics and health information technology to guide customization of molecular, clinical, and environmental information toward optimizing SUD-prevention in youth. Findings from such research may also guide precision medicine through systematic identification of risk/protective factors, biomarkers, and individual variations in these, which critically mediate effects of substance use on the trajectory of the developing brain, memory, and other cognitive areas in youth.

## Conflict of Interest Statement

The author declares that the research was conducted in the absence of any commercial or financial relationships that could be construed as a potential conflict of interest.

## References

[1] Collins FS Varmus H. A new initiative on precision medicine. N Engl J Med (2015) 372:793–5. 10.1056/NEJMp1500523 25635347 PMC5101938

[2] Precision Medicine Initiative [Internet]. Bethesda, MD: National Institutes of Health (US) (2015). Available from: http://www.nih.gov/precisionmedicine/

[3] Ghitza UE Needed relapse-prevention research on novel framework (ASPIRE model) for substance use disorders treatment. Front Psychiatry (2015) 6:37 10.3389/fpsyt.2015.00037 25798112 PMC4351566

[4] Ghitza UE ASPIRE model for treating cannabis and other substance use disorders: a novel personalized-medicine framework. Front Psychiatry (2014) 5:180 10.3389/fpsyt.2014.00180 25538635 PMC4258994

[5] Adolescent Brain Cognitive Development (ABCD) Study – Research Project Sites (U01) [Internet]. Bethesda, MD: National Institutes of Health (US) (2015). Available from: http://grants.nih.gov/grants/guide/rfa-files/RFA-DA-15-015.html

[6] Silins E Horwood LJ Patton GC Fergusson DM Olsson CA Hutchinson DM Young adult sequelae of adolescent cannabis use: an integrative analysis. Lancet Psychiatry (2014) 1:286–93. 10.1016/S2215-0366(14)70307-4 26360862

[7] Meier MH Caspi A Ambler A Harrington H Houts R Keefe RS Persistent cannabis users show neuropsychological decline from childhood to midlife. Proc Natl Acad Sci U S A (2012) 109:E2657–64. 10.1073/pnas.1206820109 22927402 PMC3479587

[8] Hall W. What has research over the past two decades revealed about the adverse health effects of recreational cannabis use? Addiction (2015) 110:19–35. 10.1111/add.12703 25287883

[9] Kelly AB Evans-Whipp TJ Smith R Chan GC Toumbourou JW Patton GC A longitudinal study of the association of adolescent polydrug use, alcohol use and high school non-completion. Addiction (2015) 110:627–35. 10.1111/add.12829 25510264 PMC4361375

[10] ISBER Best Practices for Repositories [Internet]. Vancouver, BC: ISBER Head Office (CA) (2015). Available from: http://www.isber.org/?page=BPR

